# National observational study to evaluate the Cleanyourhands campaign (NOSEC) in England and Wales 2004-8: a prospective ecological interrupted time series

**DOI:** 10.1186/1753-6561-5-S6-P117

**Published:** 2011-06-29

**Authors:** S Stone, NOSEC group

**Affiliations:** 1 Universirt College London, London, UK; 2 Hand hygiene Liaison Group, London, UK

## Introduction / objectives

WHO SAVE LIVES initiative offers nations a multimodal hand-hygiene intervention. England & Wales rolled out similar intervention, the Cleanyourhands campaign (CYHC) (bedside alcohol hand-rub (AHR), posters, audit & patient empowerment) to all 187 acute hospitals. We report results of independent study evaluating its national effectiveness & sustainability.

## Methods

6 questionnaires (5 voluntary, last mandatory) assessed CYHC implementation & sustainability every 6 months. Quarterly data on MRSAB, MSSAB & CDI, procurement soap & AHR, hospital type & bed occupancy collected for each hospital with data on other national infection control interventions Mixed effects Poisson regression model assessed associations between procurement & HCAI rates, testing for hospital heterogeneity.

## Results

Questionnaire response rates fell from 134 (71%) at 6 months to 82 (44%) at 30 months, rising to 167 (90%) for final mandatory one. No evidence attritional/ selection bias. Widespread early implementation bedside AHR & posters. At 36 months, 90% respondents reported CYHC a top hospital priority, with implementation of AHR, posters & audit reported by 96%, 97% and 91% respectively.

Combined soap & AHR procurement rose from 22 to 60mls/bed day. MRSAB rate fell from 1.88 to 0.91 cases/10000 beddays & CDI from 16.75 to 9.49. MSSAB did not fall.

Each extra ml/bed-day of AHR associated with 1.3% reduction MRSAB: IRR 0.987 (0.983, 0.991) p<.0001). Each extra ml soapÂ associated with 0.33% reduction CDI (IRR 0.997 [0.995,0.998] p<0.0001). Associations remained after adjusting for other variables significantly associated with reductions MRSAB & CDI: publication of Health Act & Department of Health Improvement Team visits.

## Conclusion

The CYHC appears to have been widely implemented & sustained. Strong associations found between procurement AHR/soap & reductions in MRSAB & CDI, that remained after adjustment for other variables & interventions. Campaign’s central funding & co-ordination and high profile political drive may affect its generalisability but may provide model for other countries to adopt to implement WHO SAVE LIVES initiative.

## Disclosure of interest

S. Stone Grant/Research support from GOJO.

